# Nurse Training in Gender-Based Violence Using Simulated Nursing Video Consultations during the COVID-19 Pandemic: A Qualitative Study

**DOI:** 10.3390/ijerph17228654

**Published:** 2020-11-21

**Authors:** Diana Jiménez-Rodríguez, María Teresa Belmonte García, Azucena Santillán García, Fernando Jesús Plaza del Pino, Alicia Ponce-Valencia, Oscar Arrogante

**Affiliations:** 1 Department of Nursing, Physiotherapy and Medicine, University of Almeria, 04120 Almeria, Spain; tbgarcia@ual.es (M.T.B.G.); ferplaza@ual.es (F.J.P.d.P.); 2 Department of Cardiology, Burgos University Hospital, 09006 Burgos, Spain; ebevidencia@gmail.com; 3 Faculty of Nursing, Campus de los Jerónimos s/n, Catholic University of Murcia, 30107 Murcia, Spain; aponce@ucam.edu; 4 University Centre of Health Sciences San Rafael, San Juan de Dios Foundation, Nebrija University, 28036 Madrid, Spain; oarrogan@nebrija.es

**Keywords:** COVID-19, gender-based violence, high fidelity simulation training, learning, nursing education, nursing students, qualitative research, video consultation

## Abstract

Gender-based violence (GBV) is a serious global public health problem that becomes aggravated during public health emergencies that involve quarantine measures. It is important to train nursing students on GBV, especially in vulnerable situations, such as the current COVID-19 pandemic. The purpose of our study is to explore the perceptions of third-year nursing students about simulated nursing video consultations for providing assistance to potential cases of GBV victims using a high-fidelity clinical simulation methodology. After all of the simulated scenarios were completed, 48 scripted interviews were carried out following a guide composed of four open-ended questions to facilitate in-depth discussion. A descriptive qualitative study based on the interpretative paradigm was conducted. The nursing students indicated that they improved their knowledge on GBV victim management (mainly their awareness of the problem, recognition of the role of nursing professionals, and performance of non-technical skills), although they also mentioned the need for continuous training (particularly in socio-emotional skills, interview techniques, a holistic nursing care approach, and not presupposing). This innovative high-fidelity simulation methodology allows nursing students to improve their awareness of the GBV problem, acquire a realistic view about their role in addressing GBV, and build their non-technical skills (such as active listening, communication skills, empathy, and generating confidence) required to adequately care for victims of GBV.

## 1. Introduction

Violence against women, or gender-based violence (GBV), is a global serious public health problem [1,2,3]. Almost 18% of women and girls aged 15 to 49 years who have ever been in a relationship have experienced physical or sexual violence by an intimate partner in the previous 12 months [4]. Its prevalence is significant, and it has a great impact on health services [5]. GBV is generally defined as any type of violence (physical, psychological, or sexual) against women that is commonly perpetrated by intimate male partners or ex-partners [6]. Female victims of physical, psychological, or sexual violence may suffer (often in silence) a broad range of health issues [7]. However, the situation of GBV victims becomes aggravated during public health emergencies that involve quarantine measures, such as the current COVID-19 pandemic [8,9].

All healthcare professionals, especially nurses, are frequently the first contact for these victims. Consequently, they play an essential role in GBV prevention, early identification, quality of care for the victim, and combating this worldwide public health problem [10]. In contrast, GBV detection rates are low [11], therefore, healthcare providers who are trained in GBV to adequately assist these victims are urgently needed [12]. Given the potential impact of future nurses for reducing GBV, nursing students may contribute to the prevention, identification, and intervention of GBV victims. Thus, they must be prepared to respond to this type of violence [11,13,14].

Since December 2019, the COVID-19 pandemic has expanded from Wuhan (China) [15] to a growing number of countries. Social distancing measures to ensure confinement, including quarantines, put vulnerable populations, such as GBV victims, at risk. Early data from China and several affected countries have shown that GBV, specifically domestic violence, has dramatically increased, exacerbated by the confined living conditions as a result of lockdown measures [8,9,16,17,18]. Under these conditions, women are exposed to violence in limited physical spaces, where household stress levels have increased due to the negative economic and health consequences of confinement [8]. However, the increase in GBV has already been demonstrated in other pandemics that included quarantine measures, such as during the Ebola and Zika emergencies [19], or economic crises [20]. Therefore, the adoption of effective initiatives for tackling this worldwide problem is essential. For example, the Spanish government launched an action guide aimed at women who were experiencing GBV at home during confinement due to COVID-19 [21]. Furthermore, new modalities of providing care, such as video consultations, are emerging to avoid COVID-19 infection between healthcare professionals and patients [22,23]. For example, the National Domestic Violence Hotline is offering services via online chat or texting to help GBV victims in the USA [8].

Several studies have highlighted the significant role of healthcare professionals in the prevention of GBV and assistance to these victims [24,25]. As nurses are frequently the first contact for these victims in most healthcare systems, they are essential for GBV prevention, early identification, and management [26]. In contrast, several studies have demonstrated that nurses are not adequately prepared to identify symptoms of violence or to care for these women [10,27]. According to WHO recommendations, all healthcare professionals should be trained to adequately identify and assist GBV victims [14]. In addition, these recommendations emphasize the need for early recognition and search of support systems for these victims [5]. Therefore, specialized teaching and training strategies on GBV are needed for healthcare professionals [28].

In Spain, most nursing education plans include specialized training in GBV in their curricula, although the topics covered tend to be heterogeneous [29]. Nevertheless, nursing education is the first step for providing a response to GBV. Since clinical care of GBV victims is complex, training interventions are needed to reduce the gap between theory and clinical practice [30]. However, the programs implemented must be rigorously evaluated to verify their correct development and the improvement of care for abused women [28].

A recent systematic review on effective educational strategies on GBV included active methodologies, such as single session lectures or seminars, interactive online tutorials, standardized patient interactions, peer education, theater, group discussion, role play, and a full-day interactive workshop covering theory and practice [31]. This review suggested that interactive educational strategies were better than theory-based strategies, as they focused on practical application for learning [31]. In this sense, a high-fidelity clinical simulation methodology accomplishes this requirement. Our study was based on the theoretical foundation of clinical simulation, as its methodology is considered an adequate strategy for training and evaluating health sciences students [32,33], and is an effective method for the improvement of acquisition of competencies [34]. Furthermore, this methodology facilitates the achievement of teaching contents, helps with the detection of educational deficiencies, and promotes the integration of knowledge and clinical skills [35]. In addition, clinical simulation methodology encourages students to reflect on aspects to be improved, creating awareness about what they need to learn and do to improve their future clinical practice [35,36]. Although several studies have validated its use with positive results, clinical simulations have been mainly carried out for training physicians in cases of sexual aggression [37,38,39]. However, there is only limited research that has evaluated simulation as a teaching methodology to prepare nursing students to assess patients experiencing GVB and intervene. These studies have been mainly focused on intimate partner violence using standardized patients [40,41]. Consequently, there is a knowledge gap on the use of high-fidelity clinical simulation for training nursing students about GBV.

Therefore, we believe that it is important to teach and train nursing students on GBV using high-fidelity clinical simulations in the shape of video consultations, especially in situations of vulnerability, such as during the current COVID-19 pandemic. We implemented this methodology to train our nursing students on other clinical situations, and they expressed a high level of satisfaction and positive perceptions about this innovative proposal [42,43]. In this way, the purpose of this research is to explore the perceptions and opinions of third-year nursing students about the use of simulated video consultations to assist potential cases of GBV victims.

## 2. Materials and Methods

### 2.1. Study Design

A descriptive qualitative study based on the interpretative paradigm [44,45] was carried out to explore the nursing students’ perceptions of a simulated clinical case of GBV, including perceived benefits and suggestions for improvement, and to analyze their opinions on the knowledge and skills acquired from the simulation. Lastly, the 32-item checklist of the consolidated criteria for reporting qualitative research (COREQ) was adopted [46].

### 2.2. Sample and Setting

The study participants were third-year undergraduate students enrolled in the four-year nursing degree at a public university in Spain (University of Almeria). Participation in the study was offered to all students and its acceptance was voluntary. Fifty-nine students participated in the mandatory high-fidelity simulation sessions. Of these, 48 students agreed to be voluntarily interviewed (81.3% response rate). The study was carried out between 26 March and 2 April 2020.

### 2.3. High-Fidelity Simulation Procedure

A high-fidelity clinical simulation scenario depicting a potential case of GBV during COVID-19 confinement was performed in the form of a simulated video consultation. This methodology is considered as one more online high-fidelity simulation teaching option [42,43]. Therefore, it was carried out using a virtual platform for online video conferences provided by the university, namely Blackboard Collaborate LauncherTM. It should be noted that the standardized patient who played the role of a potential GBV victim was a woman who was selected and trained for role portrayal to ensure a standardized process and a high level of fidelity experience [47]. The students’ performances during these simulated video consultations were evaluated according to the nursing activities related to the appropriate NIC (nursing interventions classification) interventions [48] for the resolution of each simulated clinical case of GBV (Table 1). The NICs and their related nursing activities were used because they represent the criteria needed to standardize nursing care. In this sense, both NICs and nursing activities served as a guide to discuss students’ performances during the debriefing phase. While the students were familiar with the NICs and their related nursing activities from previous course coverage on the subject, they were not informed in advance that their performance would be evaluated on this basis. The main learning objective of this methodology was for student participants to provide care for adequately managing a potential case of GVB. All simulation sessions followed the INACSL Standards of Best Practice: SimulationSM [36,49]:

- The students were divided into operational work teams (2–3 students) to perform the video consultation scenario. Consequently, a total of 20 simulated video consultations in the shape of video conferences were completed, ensuring that each student performed a simulated scenario.
- Pre-briefing: Two weeks before the performance of the simulated scenario, all students were pre-briefed in order to establish a psychologically safe context [50,51] and to consolidate the learning process [51]. A short informational document about the simulated scenario was provided to all students during the pre-briefing session, allowing them to research and plan an evidence-based approach in advance. Students were advised in advance that GBV should be a focus of their reading prior to the simulation. They had received previous coverage on the issue of GBV during the curriculum and before the intervention. This information included medical history details and health conditions of the potential GBV victim (frequent medical consultations, headaches, stress due to confinement, tiredness, inadequate nutrition, lack of sleep, and feelings of guilt and low self-esteem).
- Briefing: Brief background information was presented to contextualize the simulated clinical scenario.
- Simulated scenario: The scenario was as realistic as possible, and it simulated a video consultation to assist a potential GBV victim confined at home during the COVID-19 pandemic. The patient discloses a worsening of her anxiety disorder, and during the development of the scenario, it was necessary for the students to manage her anxiety and provide her with emotional support. These interventions are known to be important for the early detection and/or management of GBV [52]. In this way, the specific nursing activities included in the NICs were selected to work cross-sectionally on a potential case of GBV. However, all of the nursing activities and NICs only served as a guide, as the students’ performance evaluation was more extensive; during the debriefing phase, the students were encouraged to undertake in-depth reflection and analysis of positive actions, mistakes, and needed improvements detected during the simulated scenario (with feedback from nurse lecturers trained in the clinical simulation methodology). The students who provided assistance to the simulated GBV victim in real-time and the standardized patient at home who played the role (a nurse lecturer) had an operating camera and microphone in their computers during the simulated scenario.
- Debriefing: The simulated scenario was discussed after it ended [36]. The debriefing with a good judgment approach [53] was employed as the debriefing method, and the gather, analyze, and summarize (GAS) debriefing tool [54] was used to structure this debriefing phase. During this phase, the students discussed the evidence-based best practices related to the scenario. This phase was guided by nurse lecturers who had an active role in the discussion of what went well, what needed improvement, and the evidence-based approaches.

### 2.4. Data Collection

Data collection was carried out through semi-structured interviews following a guide composed of four open-ended questions to facilitate the in-depth discussion of all relevant topics. These interviews were conducted and designed by the research members of this study according to the theoretical categories related to the theoretical foundation of the debriefing phase in clinical simulation methodology. This phase encouraged students to reflect on their aspects to be improved, creating awareness about what they needed to learn and do in order to improve their future clinical practice [35,36,53,54]. The interview guide was created according to the data collection instruments employed in a previous study aimed at a simulated video consultation design for nursing students [42]. Specifically, these questions were: What went well during your performance?; What did not go so well during your performance?; What have you learned after your performance?; and What clinical skills do you consider you should continue to improve to face a real situation? All students who agreed to participate in the study were interviewed via telephone during the four days following the last simulation session. The interviews lasted 15 min on average, and the interviewer was a research team member who did not participate in the simulation sessions, thereby avoiding potential bias. Since our purpose was to capture the students’ experiences more accurately and reliably after completing the debriefing phase, it was not necessary to repeat any interview. An ad hoc interview registry was created to collect the teaching needs identified and the main results obtained during the process. The interviewer was a female nurse lecturer and researcher with extensive experience in qualitative research and clinical simulation. To minimize potential research biases, a detachment strategy was applied to promote a relativistic approach to the students’ perceptions that did not match our perceptions as social observers, and the use of inter-subjectivity facilitated an adequate understanding of the meanings provided by the students [44]. All of the interviews were recorded with the participant’s verbal consent. Lastly, a pilot study was not necessary, as indicated in a similar study that was previously conducted to evaluate the nursing students’ perceptions in other clinical situations [42].

### 2.5. Data Analysis

Qualitative data were obtained from the students’ responses to the four open-ended questions provided. A qualitative description methodology allowed us to understand the participant’s simulated experience through these questions. In this way, an explanatory description of the students’ perceptions about the experience was obtained.

All of the perceptions and opinions from the students’ interviews were transcribed and reviewed by two researchers. A content analysis of the qualitative data was performed. All of the data were stored, managed, classified, and organized using the qualitative data analysis software ATLAS.ti 8 (Scientific Software Development GmbH, Berlin, Germany). Data encoding was performed by the researchers from this study, identifying categories, separating data, encoding subcategories that emerged from the analysis process, discarding data that provided redundant information, and reviewing data again to find new emergent subcategories [45]. The categories identified were initially aligned with the four open-ended questions asked and were based on the clinical simulation methodology, but subsequently, related subcategories emerged inductively to describe the students’ perceptions. Consequently, the emergent subcategories were saturated during the analysis process, mainly in the interview technique subcategory within the skills to be improved category.

### 2.6. Ethical Considerations

Ethical approval was obtained from the Research and Ethics Board of the Department of Nursing, Physiotherapy, and Medicine at the University of Almeria (no. EFM-26/19). This research study was carried out following standards and recommendations of the international Declaration of Helsinki [55]. Before interviewing all participants, informed consent was obtained from them, explaining in detail the study purpose and data custody by the main researcher. All interviews were anonymized for encoding, analysis, and result presentation. Therefore, the participants were numerically labeled in chronological order according to the date of the interview, preceded by the letter S (student).

## 3. Results

A total of 48 nursing students participated in the study. Most of the participants were women (75%), with a mean age of 24 (mean = 24.40; SD = 8.819).

The present study analyzed matters related to performance in the simulated scenario (positive aspects and aspects about patient care to be improved upon), the learning acquired after the clinical simulation session, and the skills that should be improved upon to create high-quality healthcare for GBV. A summary of the subcategories identified following content analysis, aligning with the four questions asked to students, are presented in the following table (Table 2).

The four main categories that emerged from the four open-ended questions and their 14 corresponding subcategories supported by the participants’ narratives are described in detail below.

### 3.1. Category 1: Positive Aspects

This category was related to the students’ reflections on the aspects they believed they had performed well during the simulated scenario. The subcategories identified within this category were: active listening, encouraging the woman to overcome her anxiety, eliminate guilt from the victim, and offer available resources.

#### 3.1.1. Subcategory 1.1: Active listening

Most of the students referred to their active listening as a positive aspect. They considered that they actively listened to the patient during the simulated scenario:

“We identified that something was wrong and we strengthened our active listening, trying to empower the woman”.

(S2)

“At first, we focused on active listening and emotional support. Subsequently, through open-ended questions, we inquired about the possibility that she was experiencing gender violence”.

(S6)

“We believe that we were available to the woman all the time, using good active listening”.

(S26)

#### 3.1.2. Subcategory 1.2: Encouraging the Woman to Overcome Her Anxiety

The students highlighted providing the woman with the necessary tools to control and reduce her stress during the simulated video consultation:

“We talked with her about relaxation techniques that could serve her best”.

(S21)

“Alternative methods for treating anxiety were explained”.

(S24)

“Non-pharmacological measures were given to treat anxiety”.

(S26)

#### 3.1.3. Subcategory 1.3: Eliminate Guilt from the Victim

During her performance, the woman expressed having feelings of guilt, and many students indicated that being able to recognize and help to alleviate guilt was a positive aspect of their response:

“She was made to see that the fault was not hers”.

(S5)

“The guilt she felt was eliminated, and she was made to see that she was not at fault”.

(S15)

“The myths surrounding GBV were eliminated, and explained to her, indicating the possible future consequences and that she was not responsible for what was occurring”.

(S22)

#### 3.1.4. Subcategory 1.4: Offer Available Resources

It was highlighted that during the intervention in this scenario, the students informed the woman about the public health resources available, at the same time expressing their availability:

“Support was offered and a great amount of resources that could be used against abuse, total trust and confidentiality were also offered, calming and informing that her partner did not have to become aware. Her rights were explained to her”.

(S21)

“Information was provided about what to do if she felt that she was going through a case of GBV, the telephone number, app, etc.”.

(S16)

“Supports she can trust were offered”.

(S4)

### 3.2. Category 2: Aspects for Improvement

This category was related to the students’ reflections on the aspects they performed the worst during the simulated scenario. The students highlighted three aspects that could be improved during the scenario: not presupposing, generate trust, and analyze the context.

#### 3.2.1. Subcategory 2.1: Not Presupposing

This aspect to be improved appeared frequently. The students presupposed that the woman was a GBV victim according to the short information provided in the pre-briefing session, instead of collecting more objective information about the clinical situation through the interview:

“Something that she did not say was assumed, and she was offended”.

(S5)

“It was assumed. The reason why it was not asked, we wanted to go too fast” .

(S7)

“We made her feel uncomfortable assuming answers that she had not provided. Incorrect presumptions were made”.

(S11)

#### 3.2.2. Subcategory 2.2: Generate Trust

The students made it clear that they did not successfully gain the woman’s trust:

“Questions that were too direct were asked, trust was not created with the patient”.

(S6)

“A therapeutic environment of trust was not created”.

(S8)

“A connection with her was not achieved and we missed taking advantage of key moments for asking, an environment of trust was not generated”.

(S10)

#### 3.2.3. Subcategory 2.3: Analyze the Context

It was noted that, in the scenario, the students had not delved into the context of the situation in which the woman lived. The students believed that they should have addressed other important issues related to the woman’s environment in order to not lose relevant information.

“Questions should have been asked about the social environment, relationship with the couple, etc.”.

(S14)

“Knowing the social support is very important, and this was not asked about”.

(S29)

“A holistic view was missing”.

(S38)

### 3.3. Category 3: Learning Acquisition

The students were also asked about the learning acquired after the video consultation session. With respect to what was learned, these were centered on three subjects: awareness of the problem, nursing professional’s role in GBV, and addressing non-technical abilities.

#### 3.3.1. Subcategory 3.1: Awareness of the Problem

The students recognized that working with GBV in the clinical simulation had served them to become aware of the importance and complexity of this social problem.

“We did not stop to think that we could get a case such as this, but it is more common than what we think, and we have to be prepared and know how to act”.

(S2)

“How complicated and especially how delicate this subject is to deal with, that we have many doubts and that is not something that one can learn like any other technique, but it is a skill that students and health professionals have to emphasize”.

(S6)

#### 3.3.2. Subcategory 3.2: Nursing Professional’s Role in GBV

After the simulation experience, the students pointed to the important role that nurses play in the detection and addressing of GBV.

“…that we as health professionals are very important when detecting this violence and we should do whatever is needed to denounce these acts and help the victim”.

(S18)

“Learn to detect that there are many types of GBV and that it could be secondary to specific problems of patients who continuously visit the health center”.

(S22)

“It is a delicate problem, but what is important is listening and being able to talk about the subject so that the patient can reflect upon it and be able to become aware about it by herself”.

(S21)

#### 3.3.3. Subcategory 3.3: Addressing Non-Technical Skills

This subcategory included all of the answers of the students that dealt with aspects they learned about GBV. These were primarily focused on non-technical skills (listening, communication skills, empathy, trust, etc.).

“Trust, confidentiality should be established, and look after the worries of the patient”.

(S3)

“How to deal with the victim so that she becomes aware that she is suffering violence”.

(S9)

“I learned how to address a person with signs in a subtle manner”.

(S15)

“To empathize with the patient, to listen, to be calm, etc.”.

(S19)

### 3.4. Category 4: Skills to Be Improved

Since the adequate management of a GBV case is quite complex, the students retrospectively identified skills and aspects of their own performances that required improvement for facing a real situation of GBV in the future. The analysis of their comments resulted in four subcategories: socio-emotional skills, interview technique, achieve a holistic approach, and not presupposing.

#### 3.4.1. Subcategory 4.1: Socio-Emotional Skills

The simulation experience helped the students to learn some socio-emotional skills. However, these skills require practice and experience, so the students considered that they should continue to train for these skills. The improvements of certain socio-emotional skills, such as communication, creating an environment of trust, and recognizing and managing the patient’s emotions, were mentioned.

“How to treat the victim, how to talk to her without her feeling offended or oppressed”.

(S3)

“I believe that communication and active listening are indispensable in these cases, and these are abilities that all health professionals should work on”.

(S5)

“Learn how to create a safe environment, be careful with what I say and how I say it”.

(S29)

“We have to work on communication skills in complex situations”.

(S37)

#### 3.4.2. Subcategory 4.2: Interview Technique

The students expressed the need to improve their techniques to interview women who are victims of this type of violence.

“How to establish a conversation with the patient and read between the lines”.

(S40)

“How to ask and inquire, formulate open-ended questions”.

(S27)

“Let the person set the conversation times without pressure and with the adequate questions”.

(S48)

#### 3.4.3. Subcategory 4.3: Achieve a Holistic Nursing Care Approach

The simulation experience allowed the students to better understand the importance of a holistic approach in nursing care of any patient, and highlighted how crucial this is in cases of GBV. In their comments, they recognized the importance of improving this comprehensive approach.

“Achieve comprehensive care and address all the aspects involved in being a victim of GBV”.

(S16)

“Greater objective assessment of the patient according to scales”.

(S38)

“You have to be aware of many aspects of the person and her surroundings”.

(S43)

#### 3.4.4. Subcategory 4.4: Not Presupposing

This was previously assessed as an aspect that should be improved upon in the scenario, and it was once again present in the skills that should continuously be worked on. The simulation experience allowed the students to recognize this trend of assuming in the situation of GBV.

“That one should never presuppose anything and that we have to work with the person’s own rhythm”.

(S27)

“I think I have to learn not to presuppose anything about the life of the patient”.

(S33)

“Pay more attention to what the patient says, not pre-judge, listen more”.

(S34)

## 4. Discussion

The simulated scenario in the form of video consultations presented to nursing students was a potential case of psychological violence against a woman who was also confined at home with her partner due to the COVID-19 pandemic. It has been previously demonstrated that pandemics that include quarantine measures increase and exacerbate GBV cases [8,19]. The simulated GBV victim in our scenario featured a case of violence in a non-explicit manner (physical discomfort, irritability, hyper-attendance, and somatization) [56]. In addition, she suffered from other symptoms related to psychological violence, such as low self-esteem, anxiety disorders [26], and inadequate nutrition [5].

Regarding the nursing students’ perceptions about their performance after the simulated scenario, they mainly pointed out the positive aspects of the development of skills related to active listening, anxiety control, elimination of guilt, and offering of resources. All of these skills were consistent with the needs expressed by GBV victims and their associated health issues identified in previous studies [56,57]. Active listening is recognized as one of the main communication skills that nurses should possess for the adequate care of these victims [52,58,59].

Among the aspects to be improved upon identified by students, our results highlighted the significance of generating trust with these patients. Previous studies have also demonstrated potential deficiencies among health professionals in generating a safe environment where women are treated with respect and genuine interest and where they feel listened to and secure [12,60]. In addition, our students expressed that they should analyze the context and not presuppose in order to improve the care of GBV victims. These issues are important, as a holistic nursing care approach requires focusing on the overall context of patients and not only on their specific needs at a given time [61]. All of the deficiencies identified above were consistent with the difficulties related to communication skills and care for GBV victims expressed by healthcare professionals and students in other studies [7,11,13,56].

Conversely, our students expressed that they had improved their ability to appropriately respond to patients experiencing GBV, so the simulation session was a beneficial learning experience. This result is congruent with studies of nursing professionals using other training methodologies (conferences and training workshops) [62,63]. More specifically, nursing students reported an improvement in their awareness of the problem, recognition of the role of nursing professionals, and performance of non-technical skills aimed at GBV management. These results are consistent with evidence that concludes that training can increase knowledge, improve self-efficacy, and help healthcare professionals to acquire the skills required to adequately manage this type of violence [26] and, consequently, develop a greater awareness of the most significant physical and emotional consequences of this phenomenon in GBV victims [60]. Our results are also similar to other studies conducted with students that underlined that training increases violence awareness [27] and knowledge about GBV [64]. Furthermore, it is significant to recognize the crucial role of nursing professionals of this type of violence, as nurses are essential for the prevention of GBV [26,52].

Although nursing students expressed having improved their communication skills using the clinical simulation methodology, they also considered that they should continue to be trained on these skills, which include socio-emotional skills, interview techniques, a holistic nursing care approach, and not presupposing. These skills are considered as basic skills for their future clinical practice with GBV victims, as their adequate care and attention implies the possession of knowledge and a broad range of skills [7,10]. Since our students recognized that GBV management is complex, they perceived the need to continue preparing to provide an adequate response to this violence, strengthening their clinical and relational skills. This result reflects findings from other studies [1,30,60].

Consequently, it should be noted that both nursing students and nurses should be properly trained to assist GBV victims, as they are frequently the first contact for these victims when they seek help in health systems [26]. However, the detection rate of GBV is still low in these services [65]. Therefore, more formal training on this type of violence is needed for both nursing students and registered nurses to improve care for these women [10,14,26,29,60,66]. Therefore, we recommend including a high-fidelity clinical simulation methodology for training undergraduate nursing students and registered nurses in GBV victim management. In addition, it has been demonstrated that positive interactions with healthcare professionals lead to greater safety, support, and self-efficacy [11].

Lastly, within teaching and training methodologies, it has been demonstrated that a high-fidelity clinical simulation generated and improved learning processes [67]. Specifically, our study could fill the existing knowledge gap related to training in GBV with this methodology according to the students’ perceptions about its use, as our results showed that a high-fidelity clinical simulation could be a good method for training nursing students on non-technical skills required for adequately caring for victims of GBV.

Considering all of the above, our study is very valuable, as it demonstrates that it is possible to improve future clinical practice through clinical skills training in a realistic environment using clinical simulation methodology, which allows learning from errors without jeopardizing a patient’s security. Furthermore, some clinical management errors were identified in this study and, consequently, its detection may improve the learning of nursing students and registered nurses.

### Limitations

Since we carried out a small-scale qualitative study, there could be limitations related to the transferability of our findings. However, this study aimed to address the nursing students’ perceptions about certain aspects of simulation sessions based on a potential GBV case, and this objective was achieved. Since students enrolled in a nursing degree are commonly women, we mainly had female participants (75%), which may have influenced their responses about the experience, as they perhaps felt more identified. In addition, nursing education plans may vary by country and be heterogeneous, so our findings may not be generalized to other contexts. Although the nursing students did not select GBV-specific nursing activities (e.g., ascertaining whether the patient is safe to talk to, whether the perpetrator is nearby, whether children are involved, etc.), these issues were discussed in the debriefing phase, since safety questions are a priority in any GBV case. In this sense, we considered that only the specific NIC related to violence against women ([6403] Abuse Protection Support: Domestic Partner) was not adequate for the resolution of our simulated scenario. It should be noted that our scenario was aimed at the early detection of a potential case of gender-based violence through non-specific symptoms and anxiety, prioritizing emotional support, and anxiety management for its resolution. By addressing these NICs, the students could transversally cover the necessary aspects to detect violence (for this reason, we carefully selected the nursing activities related to each NIC). In addition, these sessions could be extended to all nursing students for further training on GBV. Thus, more research on this topic is recommended to further analyze the usefulness of simulated video consultations.

## 5. Conclusions

Simulated nursing video consultations based on potential cases of GBV could be considered as another training methodology option for caring for victims of this type of violence. As the clinical care of these victims is complex, a clinical simulation methodology could fill the existing gap between clinical theory and clinical practice in GBV. This methodology allows nursing students to improve their awareness of this phenomenon and acquire a realistic view about their role in GBV care. In addition, this methodology improves and further promotes their acquisition of non-technical skills (such as active listening, communication skills, empathy, and generation of trust) required to adequately care for these women.

## Figures and Tables

**Table 1:** Simulated scenario, appropriate NIC (nursing interventions classification) interventions, and related nursing activities for its resolution.

Simulated Scenario	NIC Intervention	Nursing Activities
A 28-year-old patient diagnosed with anxiety disorder living with her partner. Medical history: frequent medical consultations, headaches, stress due to confinement, tiredness, inadequate nutrition, lack of sleep, and feelings of guilt and low self-esteem. A potential case of gender-based violence.	[5510] Health education	Determine the personal context and sociocultural history of personal health behavior. Identify internal and external factors that can improve or decrease motivation for practicing healthy behaviors to prevent COVID-19 infection. Determine the current health knowledge of COVID-19 infection and lifestyle behaviors of the patient. Teach strategies related to COVID-19 prevention that can be employed to deal with unhealthy or risky behaviors, rather than giving advice to avoid or change behavior. Use family and social support systems to enhance the effectiveness of lifestyle or health behavior modification.
	[5820] Anxiety reduction	Help the patient verbalize her feelings, fears, concerns, problems faced, and how she copes with them. Help the patient identify the problem that causes stress. Explore strategies that reduce stress. Encourage the patient to extend her support group to family members, therapists, or psychologists. Provide help and advice and refer to specialized counseling services, if needed.
	[5270] Emotional support	Listen to expressions of feelings and beliefs, making empathetic or supportive statements. Create a safe, comfortable, and intimate environment for the patient and develop a relationship based on mutual respect and confidence. Explore with the patient what has triggered the emotions. Help the patient to recognize feelings such as anxiety, anger, or sadness. Show confidence in the patient’s ability to cope with the situation.

**Table 2:** Comprehensive list of subcategories identified after content analysis.

	Category 1: Positive Aspects	Category 2: Aspects for Improvement	Category 3: Learning Acquisition	Category 4: Skills to Be Improved
**Subcategories**	Active listening	Not pre-supposing	Awareness of the problem	Socio-emotional skills
	Encouraging the woman to overcome her anxiety	Generate trust	Nursing professional’s role in gender-based violence	Interview technique
	Eliminate guilt from the victim	Analyze the context	Addressing non-technical abilities	Achieve a holistic approach
	Offer available resources			Not pre-supposing
